# Moving Beyond Sorry: The Acknowledge-Repair-Prevent (ARP) Framework for Colleague Apologies in Medicine

**DOI:** 10.7759/cureus.102517

**Published:** 2026-01-28

**Authors:** Thomas F Heston

**Affiliations:** 1 Medical Education and Clinical Sciences, Washington State University, Spokane, USA; 2 Family Medicine, University of Washington, Spokane, USA

**Keywords:** colleague apologies, medical hierarchy, professional accountability, restorative justice, workplace conflict

## Abstract

Patient apologies are institutionalized in medicine through training, legal protections, and institutional programs. Colleague apologies remain rare despite frequent harms from hierarchy, bullying, and dismissal. This perspective examines why institutions mandate patient apologies but ignore colleague harm, despite evidence that unaddressed workplace conflict drives turnover, worsens burnout, and fractures the communication essential for coordinated care. Studies show that a majority of clinicians report that conflicts affect care quality, with nearly half perceiving possible harm to patient survival in intensive care settings. Existing apology models restore immediate dignity but lack prevention components. The proposed Acknowledge-Repair-Prevent (ARP) framework adds concrete prevention steps as systemic quality improvement measures and emphasizes restorative over retributive justice. Implementing ARP requires addressing structural barriers, including hierarchy and ego. The framework could rebuild the internal trust that sustains patient safety culture.

## Editorial

Introduction

When clinicians err with patients, saying “sorry” upholds truth-telling, expresses compassion, and is widely viewed as an ethical obligation [1]. It shows respect and humanity toward those we serve.

But what about harms to colleagues? Administrative oversights, bullying, disrespectful comments, and failures of support injure coworkers daily, yet apologies are scarce, especially across the medical hierarchy. In my career, physicians rarely apologize to each other; administrators, in my experience, never do. This silence persists despite innumerable ways in which words and actions harm peers: arrogance stemming from subspecialty training, public dismissal of valid concerns, or abandonment in crisis.

Patient apologies are mandated and protected; colleague apologies lack equivalent support. This perspective examines the disparity, its costs, and a practical remedy: the Acknowledge-Repair-Prevent (ARP) framework.

This article was previously published as a preprint on Zenodo on December 16, 2025 [2].

The workplace toll of unaddressed harm

Healthcare conflict is prevalent and potentially damaging. In a large ICU study, over 70% of staff reported conflicts, and 70% believed recent conflicts affected the quality of care, with 44% perceiving possible harm to patient survival [3]. In a separate hospital study, clinicians judged that about 40% of described conflict situations had the potential to negatively affect patient care [4]. Bullying and other forms of workplace mistreatment are common in medicine and have been shown to cause harm to individuals and teams [5].

Examples abound. I once urgently requested an emergency subspecialty evaluation; my concern was publicly dismissed in a large group email as “stupid” and “poor judgment.” Yet when the specialist finally saw the patient hours later, she was immediately rushed off to emergency surgery, which thankfully went well. The clinical course proved I was right; the subspecialist even privately admitted it to others. Yet no public acknowledgment or apology to me followed. In another instance, a mentor failed to support me during a complex case, leaving me feeling not only abandoned but betrayed. The patient recovered, but the relationship did not.

Such incidents reflect hierarchy: subspecialists belittling primary care, administrators ignoring clinician burden, and mutual disparagement across roles. Unaddressed, these incidents erode communication and morale. They worsen burnout in an already strained field.

Why patient apologies are institutionalized but colleague apologies are not

Patient apologies are institutionalized because they work. Empathetic disclosure increases forgiveness [6], reduces malpractice claims [7], and is now legally protected in over 30 states [8,9].

Patient apology programs have been shown to reduce malpractice claims by over 30% and litigation costs significantly [7]. No equivalent data exist for colleague apologies because they remain uninstitutionalized. Success metrics for ARP would include reduced staff turnover, improved psychological safety scores, decreased repeat-harm incidents, and qualitative feedback on relationship repair. Validating these outcomes would require implementation studies, as patient litigation reduction applies to patient-facing disclosure programs and cannot be directly extrapolated to colleague apologies. Hospitals train clinicians to apologize, provide scripts, and shield apology statements from litigation. No parallels exist for colleague apologies. The liability system encourages defensiveness and emphasizes individual blame [10]. This climate divides people who need to cooperate for patient care.

Healthcare institutions invest heavily in patient safety culture yet ignore the internal trust that sustains it. Unaddressed colleague harm drives turnover, worsens burnout, and fractures communication. When a nurse hesitates to escalate concerns after being publicly dismissed, or a resident withdraws after administrative abandonment, patient care suffers. The same conflict data showing potential harm to patient survival [3,4] shows that repairing colleague relationships isn't separate from clinical outcomes; repairing relationships makes the coordination of complex medical care possible. Institutions that formalize colleague apologies invest in the relational infrastructure that keeps teams functional under pressure.

The acknowledge-repair-prevent framework

Effective apologies typically require genuine remorse, specific acknowledgment, and amends [11]. These restore immediate dignity but often ignore systemic roots or future prevention. The harmed party gains validation, yet no assurance others will be spared.

The ARP framework proposed here builds on existing models by adding a prevention component and emphasizing restorative over retributive justice. Retributive dynamics prioritize status and power; restorative justice focuses on shared values, heals relationships, and rebuilds trust [12]. 

Acknowledge

Own the specific harm fully and sincerely, without deflection. “I publicly dismissed your urgent referral as poor judgment. You were correct, and my response was disrespectful.”

Repair

Offer meaningful restitution matched to the harm. Because the dismissal was public, repair could include a direct, visible correction: “In our next team meeting, I will openly acknowledge that your referral was spot-on and thank you for advocating for the patient. I will also offer to cover one of your urgent consults this month as a tangible gesture of support.”

Prevent

Commit to concrete changes preventing recurrence. “I will implement a rapid-response protocol for urgent referrals and lead team training on respectful communication.” Prevention shows the harmed person that their experience mattered and will stop future harm. The Repair step must restore social standing and psychological safety, not merely balance workload. In the example above, the public acknowledgment restores credibility among peers who witnessed the dismissal; the consult offers signals relational investment, not transactional exchange. Repair should be visible to those who witnessed the harm, proportionate to the injury, and explicitly focused on relationship restoration rather than workload compensation.

Barriers and pathways forward

Ego, insecurity, and organization charts block colleague apologies. Healthcare's litigious climate reinforces defensiveness. ARP addresses these barriers by making apologies concrete and action-oriented rather than vague expressions of regret. The prevention step reframes mistakes as system improvements, reducing ego threat. When the person apologizing has lower institutional authority, the Prevent step adapts to their sphere of control. For example, a resident who dismissively interrupted a nurse during rounds could personally commit to using a structured handoff communication tool and explicitly inviting corrective feedback. Solutions include leadership modeling ARP, training that incorporates colleague scenarios alongside patient scenarios, and lowering administrative layers that encourage posturing. Ethical implementation requires that participation be voluntary, that individuals are protected from retaliation, and that ARP supplements, rather than replaces, formal HR, professionalism, or patient-safety reporting pathways for severe misconduct.

To keep ARP usable, measurement should be minimal: (1) a single checkbox documenting whether ARP was completed for a flagged colleague-harm event, and (2) a quarterly one-item speaking-up pulse measure (Table 1). More detailed tracking (e.g., repeat-harm trends) can be reserved for mature implementations or periodic audits. When admitting mistakes becomes normal, vulnerability stops feeling risky.

**Table 1:** Metrics for ARP Implementation ARP: Acknowledge–Repair–Prevent

Measure (Minimum Viable)	Data Source
ARP completed for a flagged colleague-harm event (Yes/No; date)	One checkbox in existing incident/professionalism log
Quarterly 1-item speaking-up pulse (1–5)	Anonymous staff pulse survey

Conclusion

Medicine has built systems to apologize to patients while leaving harm within teams largely unmanaged. ARP provides a pragmatic way to close that gap: acknowledge the specific harm, repair it visibly and proportionately, and prevent recurrence. Implemented with straightforward metrics and clear ethical boundaries, ARP reframes apology as relationship-focused quality improvement. Colleague apologies deserve institutional support because team trust is not separate from patient safety; it is one of its prerequisites.
